# Date Palm Pollen (*Phoenix dactylifera* L.) Phytoestrogens as Natural Modulators of Estrus in Goats: A Molecular and Phytochemical Insight

**DOI:** 10.3390/molecules31050898

**Published:** 2026-03-09

**Authors:** Amr Kchikich, Anass Ben Moula, Ayoub Kounnoun, Said Barrijal, Mohammed El Maadoudi, Nathalie Kirschvink, Youssef Chebli, Samira El Otmani, Bouchra El Amiri, Naoual Alahlah, Mouad Chentouf

**Affiliations:** 1 Department of Biology, Abdelmalek Essaâdi University, Tangier 90000, Morocco; s.barrijal@uae.ac.ma; 2 Regional Center of Agricultural Research of Tangier, National Institute of Agricultural Research, Rabat 10090, Morocco; youssef.chebli@inra.ma (Y.C.); samira.elotmani@inra.ma (S.E.O.); 3 Laboratory of Natural and Economic Resources for Sustainable Development, Department of Life Sciences, Polydisciplinary Faculty of Larache (FPL), Abdelmalek Essaâdi University, 745 BP, Larache 92004, Morocco; a.benmoula@uae.ac.ma; 4 Regional Laboratory for Analysis and Research, National Office for Food Safety, Tangier 90000, Morocco; a.kounnoun@gmail.com (A.K.); mohammed.elmaadoudi@onssa.gov.ma (M.E.M.); naoual.alahlah@yahoo.fr (N.A.); 5 Department of Medicine, Namur Research Institute for Life Sciences (NARILIS), University of Namur, 5000 Namur, Belgium; nathalie.kirschvink@unamur.be; 6 Animal Production Unit, Regional Center Agricultural Research of Settat, National Institute for Agricultural Research (INRA), Avenue Ennasr, P.O. Box 415 Rabat Principal, Rabat 10090, Morocco; bouchra.elamiri@inra.ma

**Keywords:** 17-β estradiol, coumestrol, date palm pollen (*Phoenix dactylifera* L.), estrogen receptor alpha, molecular docking, quercetin, selective estrogen receptor modulators

## Abstract

Reproductive management in goats remains challenging due to seasonal breeding and the use of hormones that raise concerns about immunogenicity, cost, sustainability, and animal welfare. In this study, we evaluated date palm pollen (*Phoenix dactylifera* L.) (DPP) as a natural source of estrogenic compounds capable of modulating reproductive function. DPP was extracted using methanol, ethanol, acetone, and hexane, and the extracts were analyzed by ultra-performance liquid chromatography. Quercetin and coumestrol were detected in the methanolic and ethanolic extracts at comparable levels (quercetin 0.043–0.044 mg/g; coumestrol 0.987–1.015 mg/g of extract) (*p* > 0.05). The acetone extract contained significantly lower concentrations (quercetin 0.017 mg/g; coumestrol 0.033 mg/g of extract), while the hexane extract showed no detectable amounts. Molecular docking using the crystallographic structure of estrogen receptor alpha (PDB:6PIT) showed that both compounds interact with key residues of the receptor’s ligand-binding domain. Coumestrol exhibited the highest affinity (−9.3 kcal/mol), surpassing 17-β estradiol (−8.9 kcal/mol), forming several hydrogen bonds and hydrophobic contacts. Quercetin showed a lower affinity (−7.2 kcal/mol) but maintained stabilizing interactions compatible with partial agonist activity. Overall, methanol and ethanol were the most effective solvents for extracting phytoestrogens from DPP, and the findings support their potential as natural alternatives to hormones for estrus induction in goats.

## 1. Introduction

Reproductive efficiency is a key factor for the survival of small ruminant production that is economically viable, particularly in goats where reproduction may be seasonal and limited estrus expression might lead to lowered yields. In systems that are extensive and semi-intensive, reproductive management is usually based on the control of estrous cycles so as to improve fertility and coordinate mating with market and environmental conditions [1]. It is generally accepted that the use of progesterone-releasing devices, equine chorionic gonadotropin (eCG), and prostaglandin as the main components of the hormonal protocols for the induction or synchronization of estrus has been the prevalent method. However, their effectiveness is highly dependent on season, breed, and physiological status [2]. Repeated use of exogenous gonadotropins such as eCG may also induce the formation of anti-eCG antibodies, reducing fertility over time [1,2]. These biological limitations are compounded by ethical, regulatory, and economic concerns, prompting interest in safer and more natural alternatives to support reproductive performance [3].

There are several candidate phytoestrogens from plants that have similarities to estrogens, such as flavonoids, isoflavones, lignans and phenolic acids, and can interact with estrogen receptors to exert agonist or antagonist effects [4,5]. Some studies have also shown that follicle development and ovulation are due to the increase and release of follicle-stimulating hormone (FSH), luteinizing hormone (LH), and estradiol through phytoestrogens [3,6,7]. Moreover, some plant-derived chemicals like coumestrol, quercetin, and apigenin are specifically structured to trigger estrogen receptor modulators [4,8]. Besides these, the compounds have also been reported to have other traits, e.g., antioxidant activities and high availability, which make them very effective in hormone-free reproductive technologies.

The date palm pollen (*Phoenix dactylifera* L.) (DPP) is one of the most comprehensively researched natural sources of these compounds [6,7,9,10]. It is traditionally used in Middle Eastern and North African medicine to treat fertility disorders in both men and women [4]. The recent study confirmed the components of its phytochemical (quercetin, apigenin, caffeic and chlorogenic acids, saponins, phytosterols (b-sitosterol), and even estrone and estradiol) [4,8].

Furthermore, it has already been shown that pure pollen contains more polyphenols, flavonoids, and essential nutrients than mixtures containing spadix fragments [9]. In animals, DPP supplementation has been associated with increased gonadal weight, follicular development, and elevated serum concentrations of FSH, LH, and estradiol [3,6,7]. The biological effects of these molecules may act through direct effect on estrogen receptors, particularly the estrogen receptor alpha (ERα), which is involved in the regulation of reproductive cycle hormones. ERα modifies its conformation and activates the transcription of target genes when bound to 17-β-estradiol [5].

As DPP phytoestrogens such as yamogenin acetate, diosgenin [11], catechin, epicatechin, daidzein and quercetin [12], are structurally similar to estradiol, they have been proposed as ERα ligands [4,8]. These interactions can be predicted and analyzed in silico using molecular docking simulations, providing insight into ligand binding affinity, the nature of interactions with ERα amino acids, and overall fit with the receptor [5]. These results guided the objective of the present study, which was to identify and measure phytoestrogenic compounds in DPP extracts collected with methanol, ethanol, acetone, and hexane, using ultra-performance liquid chromatography (UPLC), and to determine their affinity for ERα using the molecular docking method. An anchoring model was based on the crystallographic structure of the oestradiol receptor (PDB ID: 6PIT), with 17-β-estradiol as the reference ligand.

## 2. Results

### 2.1. Molecular Docking

**Figure 1:**
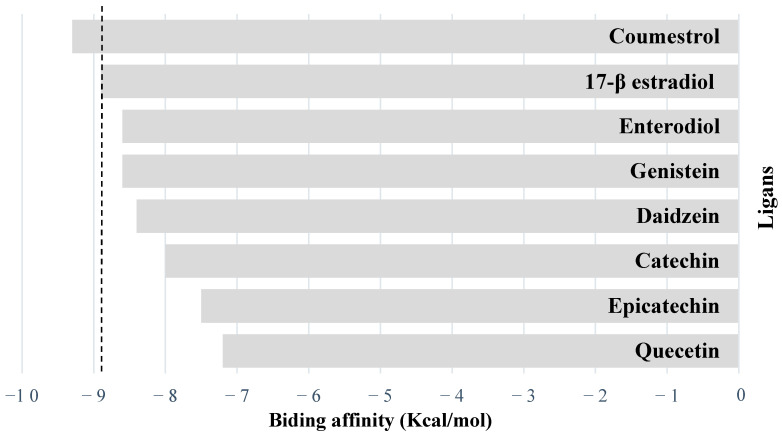
Binding affinities (kcal/mol) of selected phytoestrogens and 17-β estradiol with the estrogen receptor alpha (ERα, PDB: 6PIT). Values correspond to the predicted binding energy of the top-ranked docking pose returned by AutoDock Vina.

Molecular docking simulations were performed between selected phytoestrogens and the ERα using the crystallographic structure 6PIT. Docking poses were considered valid with root mean square deviation values < 2 Å relative to the co-crystallized reference ligand, confirming the reliability of the interaction models. The binding affinities (kcal/mol) obtained from AutoDock Vina 1.2.7 are summarized in Figure 1.

Among all tested compounds, two lignans, coumestrol and enterolactone, exhibited the strongest binding to ERα (−9.3 kcal/mol), surpassing the natural agonist 17-β-estradiol (−8.9 kcal/mol). Moderate binding affinities were observed among several isoflavones, including genistein and enterodiol (−8.6 kcal/mol each), and daidzein (−8.4 kcal/mol), with interaction strengths comparable to the endogenous ligand. In contrast, flavonoids such as Catechin (−8.0 kcal/mol), Epicatechin (−7.5 kcal/mol), and quercetin (−7.2 kcal/mol) showed weaker binding energies. Finally, the steroidal saponins yamogenin acetate (−7.3 kcal/mol) and diosgenin (−6.8 kcal/mol) displayed the lowest affinities among all tested compounds, indicating limited compatibility with the ERα binding pocket.

#### Type of Interaction

**Figure 2:**
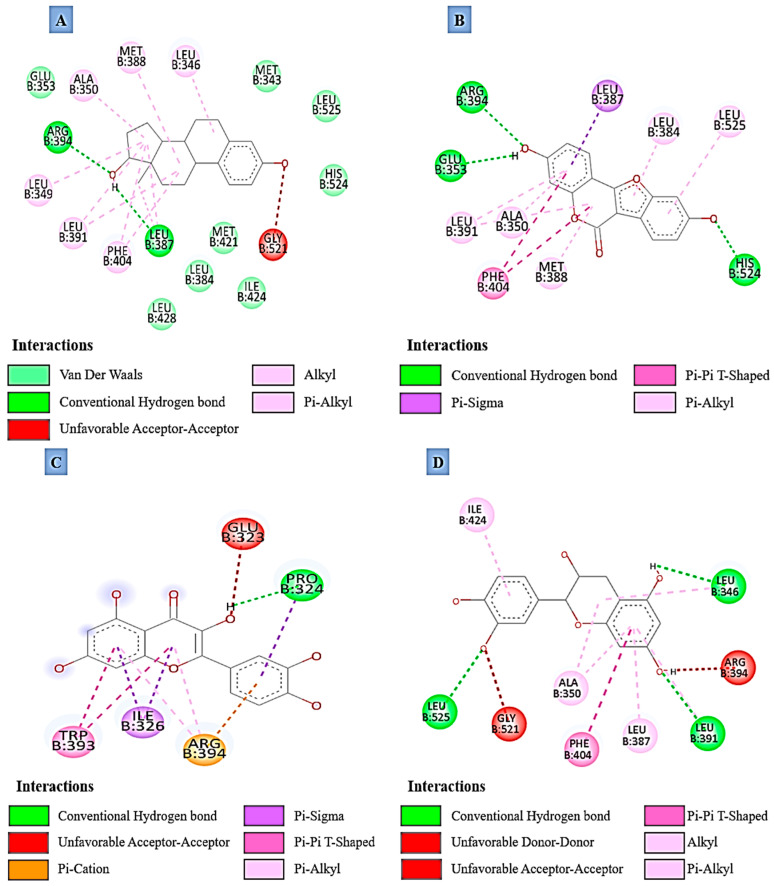

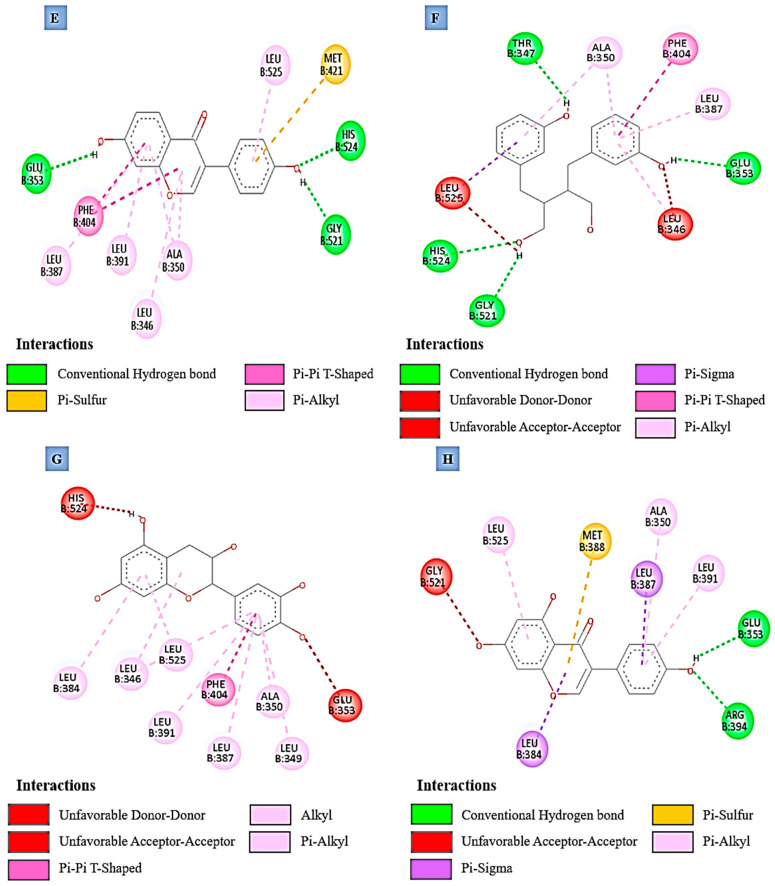
Two-dimensional interaction diagrams of 17-β estradiol (**A**), coumestrol (**B**), quercetin (**C**), catechin (**D**), daidzein (**E**), enterodiol (**F**), epicatechin (**G**) and genistein (**H**) with the estrogen receptor alpha (ERα, PDB: 6PIT). All interaction diagrams and chemical structure renderings were generated using Discovery Studio Visualizer 2025 (BIOVIA).

The molecular interaction profiles of 17-β estradiol (agonist) and quercetin and coumestrol (present in DPP extract) with the ERα revealed distinct binding patterns as illustrated in Figure 2. 17-β estradiol forms two conventional hydrogen bonds with ARG B:394 and LEU B:387, along with multiple hydrophobic interactions including π-alkyl contacts with PHE B:404, ALA B:350, MET B:388, and LEU B:346, and alkyl contacts with LEU B:349, LEU B:391, and LEU B:387. Several van der Waals interactions are also observed with GLU B:353, LEU B:428, LEU B:384, MET B:421, ILE B:424, MET B:343, LEU B:525, and HIS B:524. One unfavorable acceptor–acceptor interaction occurs with GLY B:521. Coumestrol forms three conventional hydrogen bonds with ARG B:394, GLU B:353, and HIS B:524. It also interacts via π-sigma contact with LEU B:387, π–π T-shaped stacking with PHE B:404, and π-alkyl interactions with LEU B:384, LEU B:525, ALA B:350, MET B:388, and LEU B:391. Quercetin engages in a single conventional hydrogen bond with PRO B:324, and displays π-cation interaction with ARG B:394, π-sigma contacts with ILE B:326 and PRO B:324, π–π T-shaped stacking with TRP B:393, and a π-alkyl interaction with ARG B:394. An unfavorable acceptor–acceptor interaction is detected with GLU B:323.

Catechin forms three conventional hydrogen bonds with LEU B:525, LEU B:391, and LEU B:346, and displays π-alkyl interactions with ALA B:350, LEU B:387, LEU B:346, and LEU B:391, along with an alkyl contact involving ILE B:424. A π–π T-shaped stacking interaction is also observed with PHE B:404. One unfavorable acceptor–acceptor interaction occurs with GLY B:521, and one unfavorable donor–donor interaction with ARG B:394. Daidzein forms three conventional hydrogen bonds with GLU B:353, HIS B:524, and GLY B:521, and exhibits multiple hydrophobic contacts including π-alkyl interactions with LEU B:387, LEU B:391, LEU B:346, ALA B:350, and LEU B:525. A π–π T-shaped stacking interaction is detected with PHE B:404, together with a π-sulfur interaction involving MET B:421. Enterodiol forms four conventional hydrogen bonds with THR B:347, GLU B:353, HIS B:524, and GLY B:521, and shows π-alkyl interactions with ALA B:350 and LEU B:387, a π-sigma interaction with LEU B:525, and a π–π T-shaped stacking interaction with PHE B:404. One unfavorable acceptor–acceptor interaction occurs with LEU B:346, and one unfavorable donor–donor interaction with LEU B:525. Epicatechin exhibits several hydrophobic interactions including π-alkyl contacts with ALA B:350, LEU B:387, LEU B:525, LEU B:346, and LEU B:384, as well as alkyl contacts with LEU B:349 and LEU B:391. It also forms a π–π T-shaped stacking interaction with PHE B:404. One unfavorable acceptor–acceptor interaction is observed with HIS B:524, and one unfavorable donor–donor interaction with GLU B:353. Genistein forms two conventional hydrogen bonds with GLU B:353 and ARG B:394, and displays π-alkyl interactions with LEU B:525, ALA B:350, and LEU B:391. π-sigma contacts involving LEU B:387 and LEU B:384 are also detected, together with a π-sulfur interaction with MET B:388. One unfavorable acceptor–acceptor interaction is observed with GLY B:521.

### 2.2. Phytoestrogen Composition of Date Palm Pollen (Phoenix dactylifera *L.*)

**Figure 3:**
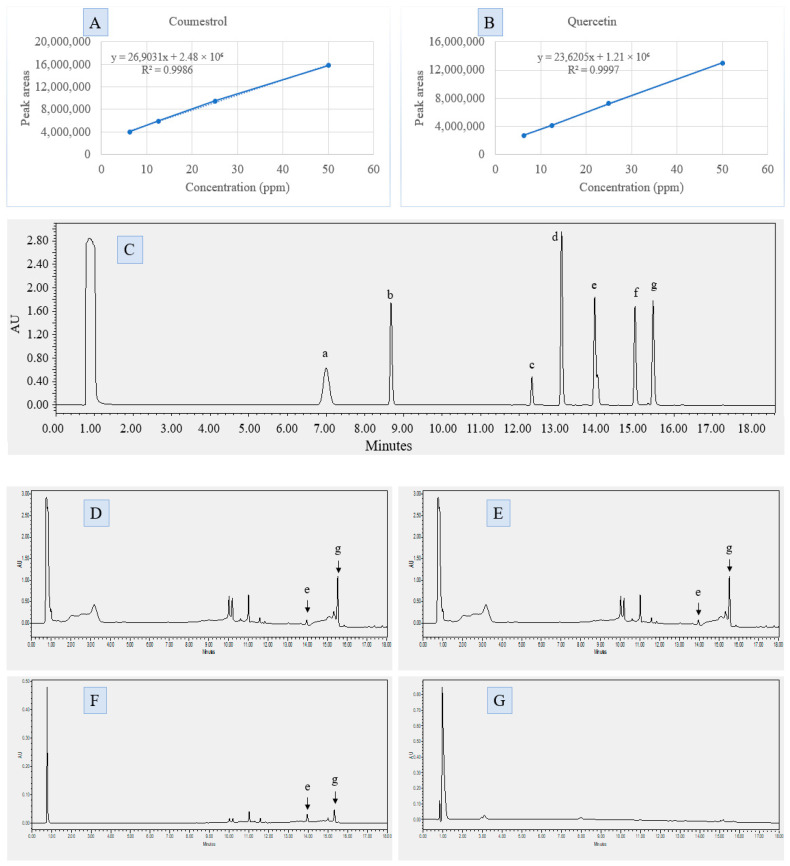
(**A**) Calibration curve of coumestrol and (**B**) quercetin, each prepared using standard solutions at 6.25, 12.5, 25, and 50 ppm with R^2^ values of 0.999 and 0.999, respectively. (**C**) UPLC chromatogram of the standard mixture, displaying retention times for catechin (a), epicatechin (b), enterodiol (c), daidzein (d), quercetin (e), genistein (f), and coumestrol (g). (**D**–**F**) UPLC chromatograms of the methanolic (**D**), ethanolic (**E**), and acetone (**F**) extracts of date palm pollen (*Phoenix dactylifera* L.), all showing peaks (e) and (g) corresponding to quercetin and coumestrol. (**G**) UPLC chromatogram of the hexane extract.

UPLC analysis of DPP extracts obtained with different solvents revealed that quercetin and coumestrol were consistently detected in the methanolic, ethanolic, and acetone extracts, while the hexane extract did not show detectable levels of these compounds (Figure 3D–G).

In the methanolic extract, quercetin and coumestrol were quantified at 0.043 ± 0.005 mg/g and 1.015 ± 0.045 mg/g of extract, corresponding to 2.157 ± 0.261 µg/g and 50.742 ± 2.25 µg/g of DPP, respectively (Table 1). The ethanolic extract yielded comparable values, with quercetin at 0.044 ± 0.005 mg/g and coumestrol at 0.987 ± 0.053 mg/g of extract, equivalent to 2.209 ± 0.275 µg/g and 49.353 ± 2.651 µg/g of DPP. Statistical comparison showed no significant difference (*p* > 0.05) between methanolic and ethanolic extracts for both compounds.

In contrast, the acetone extract contained markedly lower amounts, with quercetin at 0.017 ± 0.006 mg/g and coumestrol at 0.033 ± 0.005 mg/g of extract, corresponding to 0.852 ± 0.282 µg/g and 1.672 ± 0.263 µg/g of DPP, respectively. These values were significantly different (*p* < 0.05) from those of the methanolic and ethanolic extracts. The hexane extract did not reveal any detectable peaks of quercetin or coumestrol under the same analytical conditions. 

Other chromatographic peaks were observed in all extracts (methanol, ethanol, acetone, and hexane), unidentified as they did not correspond to the standard phytoestrogens targeted in this analysis.

## 3. Discussion

The present study identified phytoestrogenic compounds in DPP extracts obtained with methanol, ethanol, acetone, and hexane. Quercetin and coumestrol were detected in the methanolic, ethanolic, and acetone extracts, whereas hexane showed no detectable peaks under identical analytical conditions.

Methanol- and ethanol-derived extracts had comparable concentrations (quercetin 0.043–0.044 mg/g extract; 2.157–2.209 µg/g DPP; coumestrol 0.987–1.015 mg/g extract; 49.353–50.742 µg/g DPP) (*p* > 0.05). In comparison, the acetone extract had a much lower concentration of both analytes (quercetin 0.017 mg/g; 0.852 ug/g DPP; coumestrol 0.033 mg/g; 1.672 ug/g DPP; *p* < 0.05 compared to methanol/ethanol). These results suggest that the most effective solvents for extracting these phenolic phytoestrogens from DPP are polar, protic solvents (methanol, ethanol), while acetone, a polar, aprotic solvent, appears to be less effective, and hexane, a non-polar solvent, is not useful [13,14,15]. For the other targeted compounds (daidzein, genistein, enterodiol, enterolactone, (+)-catechin, and (−)-epicatechin), no detectable signals were observed, which may be attributed to the analytical method’s sensitivity, solvent selectivity, or chemotypic variation in the DPP [9,13,14]. Phytoestrogens such as quercetin and coumestrol are known to interact with ERα to regulate endocrine functions, highlighting their importance as natural alternatives to synthetic hormones in reproductive research. In previous studies, DPP is naturally rich in flavonoids (e.g., quercetin, luteolin, apigenin, isorhamnetin), phenolic acids (e.g., caffeic, chlorogenic, gallic), triterpenoid saponins, steroids, and phytosterols, e.g., estrone and b-sitosterol [4].

The antioxidant effects of DPP are enhanced by the presence of polyphenols, which are also known to increase the regulatory effects of DPP on the endocrine system [8]. Specifically, Tahvilzadeh et al., 2016 [4] identified the presence of oestradiol, oestrone and oestriol in methanolic extracts, confirming the hypothesis that DPP releases estrogen-like bioactive substances. Furthermore, Salhi et al., 2024 [10] pointed out that the origin of the cultivar and the extraction method could greatly influence the pharmacological characteristics of DPP. Clinical and in vivo experiments have confirmed the oestrogenic effects of DPP from a pharmacological point of view. Jiheel and Arrak (2015) [7] also showed that DPP ethanolic extract increased serum FSH and LH levels in mature rats, probably due to the presence of flavonoids and steroidal compounds in the extract. These results are therefore coherent with the conventional use of DPP to promote fertility and ovarian activity [10]. Overall, the discovery of quercetin and coumestrol in this research adds value and corroborates the accumulated data on the hormonal action of DPP. Its botanical composition determines its high phytoestrogen content, highlights its potential in the natural regulation of reproductive hormones, offering an increasingly desirable alternative to artificial endocrine treatments.

Estrogens primarily achieve their reproductive functions through the ERα, a nuclear receptor that is found both in the reproductive tract and in the hypothalamic-pituitary axis [16]. With the attachment of the ligand, ERα experiences changes in its shape that lead to transcriptional activation of the genes involved in GnRH release, gonadotropin (LH, FSH) secretion and, eventually, ovulation as well as follicular development [17,18]. The estrous cycle in goats is regulated by this hierarchal signaling pathway. Based on the results of molecular docking simulations, it was found that among the DPP phytoestrogens, coumestrol could interact with ERα most firmly (binding affinity of –9.3 kcal/mol) even better than the native ligand 17-β estradiol (–8.9 kcal/mol). Essentially, coumestrol established multiple stabilizing contacts with key ligand binding domain residues, in particular with ARG B:394, GLU B:353, PHE B:404, and LEU B:387 through hydrogen bonding, π–π stacking, and hydrophobic contacts [19,20]. These interactions patterns are consistent with a stable docking pose within the receptor’s active site [21], and may therefore be interpreted as supporting a mode of agonistic engagement of ERα by coumestrol, similar to that of estradiol. In contrast, the weaker affinities of catechin, epicatechin, enterodiol, quercetin, daidzein, and genistein compared with 17-β estradiol can be attributed to their less favorable interaction profiles, characterized by fewer strong stabilizing contacts such as hydrogen bonds and aromatic π–π or π-cation interactions, and a greater reliance on weaker hydrophobic contacts (π-alkyl/alkyl), which together provide lower overall binding stability within the ERα pocket [22,23]. Quercetin, another phytoestrogen present in DPP, was found to bind with the lowest affinity (−7.2 kcal/mol) and formed fewer stabilizing interactions, including one conventional hydrogen bond (PRO B:324), a π-cation interaction (ARG B:394), and π–π stacking (TRP B:393). While the moderate level of interaction reached, the particular type of interaction is enough for the receptor to achieve ligand-induced stabilization, which has been associated with physiological estrogenic activity [24]. It should also be noted that the reported binding affinities are docking scores derived from the AutoDock Vina scoring function and therefore represent approximate, comparative estimates rather than experimentally validated free energies of binding; more advanced approaches (e.g., molecular dynamics-based free-energy calculations) would be required to refine these predictions in future work.

Notably, Slighoua et al., 2023 [25] concluded that quercetin supplementation promoted serum estradiol levels and uterine weight increase, and Ma et al., 2022 [26] found diminished LH levels and estrous cyclicity recovery. These in vivo experiments can be interpreted as partial agonists of ERα action in line with their hypothesis. What is more, the fact that both coumestrol and quercetin interact with ERα suggests that they may act as selective estrogen receptor modulators. The pattern of interactions not only indicates high receptor selectivity but also suggests the ability to trigger conformational changes within the receptor that may contribute to its activation [27]. Retana-Marquez et al., 2012 [18] noted that coumestrol displays structural similarity to oestradiol, which enables the compound to bind to ERα in a competitive manner and co-activate estrogen-sensitive genes in reproductive tissues. However, it is important to bear in mind the diversity of compounds present in natural DPP extracts besides the similarity of the agonist activity of coumestrol to that of quercetin. In fact, a few flavonoids and polyphenols, such as apigenin and kaempferol, under certain concentration and cellular environmental conditions, may behave as antagonists of the ERα receptor [28]. Similarly, Pang et al., 2018 [29] discovered a number of natural molecules, including epigallocatechin gallate, ellagic acid, and phloretin, that are effective inhibitors of ERα activation in cellular experiments. By binding to estradiol at the receptor binding domain, these compounds suppress transcriptional activation. The flavonoids present in DPP also have close structural relationships with known ERα antagonists, confirming the hypothesis that the total estrogenic effect of DPP may reflect an active balance between compounds that can act as agonists and those that can counteract their action. More investigation of this possible interaction should be performed by bioassay-guided fractionation methods and functional activity tests targeting receptors. In general, the present data can be seen as a good mechanistic basis to account for DPP phytoestrogens as a viable substitute of synthetic hormones for estrus induction.

For quite a while, estrus induction and synchronization schemes for the reproductive management of small ruminants have been carried out with the aid of eCG. However, significant evidence has cast doubt on the biological and immunological effects of repeated administration of these hormones. Among the principal limitations of eCG based protocols is the production of anti-eCG antibodies after repeated treatments, which gradually reduce fertility [30]. Results obtained in small ruminants also show a loss of synchronization efficiency over time, increased variability in ovulatory responses and reduced fertility [31]. The overall physiological cost of hormonal treatments highlights the value of discovering natural bioactive compounds that can support reproductive performance with minimal side effects. In this context, phytoestrogens present in DPP, such as coumestrol and quercetin, could even be considered as a potentially interesting new source. These natural products can mimic the structure and effect of endogenous oestrogens and interact with ERα receptors without causing an immune response. Unlike exogenous gonadotropins, phytoestrogens are endocrine mediators that, thanks to their natural signaling pathways, can stimulate ovarian activity. Their availability, compatibility with the body, and good safety profile make them a very interesting alternative for estrus induction treatments, especially in low-input, environmentally friendly livestock production systems.

To translate these findings into practical reproductive applications, further work is needed to confirm the endocrine activity of the identified phytoestrogens under physiological conditions. Priority developments should include in vitro functional tests on ERα-sensitive ovarian or hypothalamic reproductive cell types to validate hormone-like signaling, followed by toxicological and pharmacokinetic evaluations to ensure safety and bioavailability. Ultimately, controlled in vivo trials in goats will be essential to determine optimal dosing and real estrus-induction efficacy before integration into breeding programs.

## 4. Materials and Methods

### 4.1. Experimental Design

**Figure 4:**
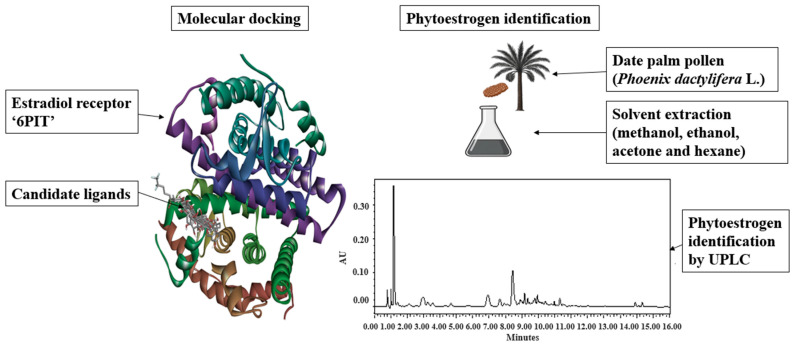
Methodological framework for evaluating phytoestrogenic components of date palm pollen (*Phoenix dactylifera* L.) as selective estrogen receptor modulators.

This study was designed to evaluate, through an in silico and analytical approach, the potential of bioactive compounds from DPP to act as selective estrogen receptor modulators with the aim of regulating the ovulatory cycle in goats. The workflow combined molecular docking to predict the affinity of candidate phytoestrogens for ERα with UPLC to identify and quantify these compounds in DPP methanol, ethanol, acetone and hexane extracts. A schematic overview of the experimental design is presented in Figure 4.

### 4.2. Molecular Docking

Among the available crystal structures of ERα, the three-dimensional structure PDB: 6PIT containing the ligand-binding domain in complex with estradiol and a coactivator peptide was used as the receptor model for the docking simulations. This structure, derived from *Homo sapiens*, is considered representative of the *Capra hircus* ERα due to the high sequence homogeneity and functional conservation of the ligand-binding domain across mammalian species [16,27]. Consequently, 6PIT serves as a structurally reliable model for goat ERα in molecular docking studies. The three-dimensional configurations of selected ligands including coumestrol, epicatechin, daidzein, quercetin, catechin, genistein, enterodiol, and 17-β-estradiol were retrieved from PubChem (https://pubchem.ncbi.nlm.nih.gov, accessed on 10 July 2024). Computational tools such as AutoDockTools 1.5.7 (Scripps Research Institute, San Diego, CA, USA), PyMOL 3.1.5.1 (Schrödinger, LLC., New York, NY, USA) [32] and Discovery Studio Visualizer 2025 (BIOVIA) [33] were used for ligand and receptor preparation and visualization of docking positions and interaction, while binding affinities (kcal/mol) were obtained using PyRx (Virtual Screening Tool) integrated AutoDock Vina 1.2.7 [34]. For each ligand, only the affinity corresponding to the highest-ranked pose returned by AutoDock Vina was used for quantitative comparison. Protein pretreatment involved removing crystallographic water molecules and irrelevant ligands or cofactors, adding polar hydrogens and assigning partial atomic charges. All molecules were converted from *.pdb to *.pdbqt format before docking. The docking grid was defined with a center at coordinates x = −3.777, y = −5.760, z = 21.807, and dimensions of 40 × 40 × 40 Å. These coordinates correspond to the crystallographic position of estradiol in chain B of ERα, and the box dimensions were chosen to encompass the ligand-binding pocket and its surrounding residues. To validate the docking protocol, the co-crystallized ligand present in the 6PIT structure was re-docked and its predicted position compared with the experimentally resolved conformation [35].

### 4.3. Extraction of Date Palm Pollen (Phoenix dactylifera *L.*)

DPP, a commercial product from Errachidia (Morocco) used for the pollination of date palm trees, was subjected to solvent extraction. For each solvent (methanol, ethanol, acetone, and hexane), 1 g of DPP was macerated in 10 mL of solvent at room temperature for 24 h [36,37]. After extraction, the mixture was filtered using Whatman^®^ (Kent, UK) filter paper to eliminate solid residues. The filtrate obtained was then concentrated by evaporation of the solvent using a rotary evaporator (Rotavapor^®^, Flawil, Switzerland) at 40 °C, a temperature selected to minimize potential degradation of thermolabile constituents. Finally, a 1 g aliquot of the extract obtained was dissolved in 10 mL of a methanol-water mixture (80:20, *v*/*v*; extra-pure grade), then filtered through a 0.45 µm syringe filter before being analyzed by UPLC.

### 4.4. Chromatographic Analysis of Phytoestrogens Using Ultra-Performance Liquid Chromatography

Phytoestrogen identification was carried out using an ACQUITY UPLC system (Waters, Milford, MA, USA) equipped with a quaternary solvent manager pump, an FTN-H sample manager, a column manager, a photodiode array (PDA) detector, and Empower 3 software for system control and data acquisition. Chromatographic separation was performed on an XBridge Shield RP18 column (150 mm × 2.1 mm, 3.5 µm particle size) coupled with an XBridge BEH Shield RP18 guard column (5 × 2.1 mm, 3.5 µm particle size). The mobile phase consisted of Solution A: ultrapure water containing 0.005 M ammonium formate and 0.01% formic acid, and Solution B: acetonitrile. The gradient elution program was defined as follows: starting at 95% A and 5% B at a flow rate of 0.500 mL/min, this composition was maintained until 5.00 min. From 5.01 to 26.00 min, Solution B was gradually increased to 95% and Solution A reduced to 5%. This ratio was held until 27.00 min, then returned to the initial conditions (95% A and 5% B) at 27.01 min and maintained until 31.00 min for column re-equilibration. The column temperature was maintained at 40 °C, and the sample compartment at 10 °C. The injection volume was 10 µL. Detection was performed using a PDA detector (Waters, Milford, MA, USA) set to scan wavelengths from 200 to 400 nm. The total runtime for each analysis was 31 min Standard compounds used for calibration and peak identification included genistein (Sigma Aldrich, St. Louis, MO, USA; 92,136), daidzein (HWI, Rülzheim, Germany; 0945), enterolactone (Phytolab, Vestenbergsgreuth, Germany; 80,437), enterodiol (Phytolab, Vestenbergsgreuth, Germany; 80,436), quercetin (Phytolab, Vestenbergsgreuth, Germany; 89,262), (+)-catechin (Phytolab, Vestenbergsgreuth, Germany; 89,172), (−)-epicatechin (Phytolab, Vestenbergsgreuth, Germany; 99,627,492), and coumestrol (Phytolab, Vestenbergsgreuth, Germany; 82,308). Calibration curves were established for each standard compound using four concentration levels (6.25, 12.5, 25, and 50 ppm). Each phytoestrogen standard was injected individually to determine its retention time and generate calibration data for identification and quantification in the DPP extract.

### 4.5. Statistical Analysis

Data are reported as mean ± standard deviation with *n* = 4 independent extractions per solvent group (ethanol, methanol, aceton and hexane). For each compound (in mg/g extract and µg/g DPP), a one-way ANOVA was performed on all solvents, followed by a Tukey HSD post hoc test. The significance threshold was *p* < 0.05. Analyses were performed in SAS 9.4.

## 5. Conclusions

This study suggests that date palm pollen (*Phoenix dactylifera* L.) may represent a natural alternative for estrus induction in goats. Ultra-performance liquid chromatography of methanolic and ethanolic extracts identified two phytoestrogens coumestrol and quercetin in similar concentrations, whereas acetone yielded much less and hexane yielded none. Both molecules can bind estrogen receptor-alpha, with coumestrol showing a stronger in silico interaction than 17-β-oestradiol. However, this difference lies within the expected uncertainty range of molecular docking scoring functions. The evidence from the studies point to the fact that substances found in date palm pollen might act as selective estrogen receptor modulators, thus imitating the natural estrogenic effects. Considering the side effects of synthetic hormones such as equine chorionic gonadotropin, the use of phytoestrogens from date palm pollen is a promising alternative solution. Nevertheless, the fact that there might be some antagonistic compounds in the extract makes it necessary to perform more in vivo and functional experiments to confirm its potency and safety for use in reproduction.

## Figures and Tables

**Table 1:** Concentration of phytoestrogenic compounds (quercetin and coumestrol) identified in DPP extracts obtained with methanol, ethanol, acetone, and hexane. Values are expressed in milligrams per gram of dry extract and micrograms per gram of DPP.

	Compound	mg/g of Extract	CV %	µg/g of DPP	CV %
Methanol	Quercetin	0.043 ± 0.005 ^A^	11.6	2.157 ± 0.261 ^A^	12.1
	Coumestrol	1.015 ± 0.045 ^a^	4.4	50.742 ± 2.25 ^a^	4.4
Ethanol	Quercetin	0.044 ± 0.005 ^A^	11.3	2.209 ± 0.275 ^A^	11.5
	Coumestrol	0.987 ± 0.053 ^a^	5.4	49.353 ± 2.651 ^a^	5.4
Acetone	Quercetin	0.017 ± 0.006 ^B^	35.3	0.852 ± 0.282 ^B^	35.2
	Coumestrol	0.033 ± 0.005 ^b^	15.1	1.672 ± 0.263 ^b^	16

Data are shown as mean ± standard deviation with *n* = 4. Different uppercase letters (^A, B^) denote significant differences (*p* < 0.05) in quercetin concentrations, and different lowercase letters (^a, b^) denote significant differences (*p* < 0.05) in coumestrol concentrations across extraction solvents, for both mg/g of extract and µg/g of DPP; CV: Coefficient of variation.

## Data Availability

Data are contained within the manuscript.

## Abbreviations

DPP	date palm pollen (*Phoenix dactylifera* L.)
eCG	equine chorionic gonadotropin
FSH	follicle-stimulating hormone
LH	luteinizing hormone
ERα	estrogen receptor alpha
UPLC	ultra-performance liquid chromatography
PDA	photodiode array
